# The perspectives of volunteers with disabilities and sport event organizers on educating and employing volunteers with disabilities

**DOI:** 10.3389/fspor.2025.1543502

**Published:** 2025-03-21

**Authors:** Marlene Jobst, Christoph Kreinbucher-Bekerle

**Affiliations:** Institute of Human Movement Science, Sport and Health, University of Graz, Graz, Austria

**Keywords:** inclusion, sport event organization, education, sport management, disability, assistance

## Abstract

**Introduction:**

Inclusive sport events can increase the social inclusion of people with disabilities. While originally, people with disabilities are represented as athletes, they could also be a valuable resource volunteering at sport events. However, barriers to their inclusion exist due to a lack of accessibility at the event venue or missing knowledge of the event organizers about collaborating with people with disabilities. Training courses for volunteers with disabilities are rare but would help to integrate them at sport events. The present study reflects on the training course and the participants' implementation in different sport events.

**Methods:**

Therefore, six so called sport management assistants with disabilities (4 female and 2 male; age: *M* = 31.67; *SD* = 6.07) and five sport event organizers (3 female and 2 male; age: *M* = 41.20; *SD* = 9.04) were interviewed with a semi-structured guideline.

**Results:**

The feedback of both groups concerning their experiences was mainly positive. The training course prepared the sport management assistants with disabilities for their tasks at the sport events. The sport management assistant graduates were satisfied with their roles at the events but criticize the lack of accessibility. The organizers praised the enthusiastic workforces added to their teams and want to cooperate with volunteers with disabilities in future events.

**Discussion:**

Thus, preparatory courses for people with disabilities as an educational tool should be encouraged to promote inclusion at sport events on a larger scale.

## 1 Introduction

The representation of people with disabilities in sport as leisure time activity is a basic human right and stated in the Convention on the Rights of Persons with Disabilities (1). Besides being physically active or an athlete participating in competitions, it is crucial that people with disabilities are also regarded as employees or volunteers in sport events (2). Otherwise, sport event organizers fail to provide them with social participation. This includes establishing relationships, cultivating contacts, improving one's social self-perception as well as being socially accepted by others despite not being able to be physically active in a competition (3). Furthermore, it elevates the level of social inclusion (4), positively impacts employment opportunities and heightens self-esteem (2). Being recruited as a volunteer allows people with disabilities to defy prejudice against them by demonstrating to people attending the event what tasks they are able to fulfil (5). Aspects related to disabilities are not as prominent (6) and they can highlight traits about their persona they want others to know about, such as being a helpful resource at a sport event. Despite usually not receiving any money for volunteering which refers to “organized voluntary roles undertaken for nonprofit or public sector organization” [(7), p. 527] at sport events, tasks at the event such as accrediting athletes and staff can enrich the lives of the volunteers as they contribute to society and are responsible for making sport events a success (8). Moreover, the tasks allow for personal growth and learning new skills for one's career. Volunteers with disabilities might enjoy their assigned activities at the event and be satisfied with the outcome (9). Generally, people with disabilities experience the effect of volunteering, for instance, enjoyment at work and the development of new skills in a similar way to other volunteers without disabilities (10). Nevertheless, there persists a need for more research regarding volunteers with disabilities and how they can be prepared for their engagement in terms of an educational process (11). While this area has been approached in different EU countries from the perspective of the event organizers (12), it has not been discussed in relation to sport assistants with disabilities yet.

Despite those prevalent positive effects of volunteering, people with disabilities face barriers to inclusion in sport event organization. They might exist on the individual level due to a lack of motivation or skills (3). But also structural aspects concerning financial resources and suitable infrastructure which prevails in a majority of German sport facilities showcasing stairs at the entrance, unorganized storage space or poor lighting are problematic (13). Environmental barriers such as policies and societal attitudes also persist (3). One of the main reasons why organizers refrain from including people with disabilities during their events is the lack of experience and knowledge surrounding the topic (2). Thus, it is vital to discuss their current role in sport event planning and the inclusion of people with disabilities as volunteers.

### 1.1 The situation of volunteers with disabilities

Volunteering refers to unpaid work to benefit others and is a worthwhile option for people with and without disabilities. Trembath et al. (14) have shown that employment plays a vital role in the life of adults. It provides them with stability, emotional well-being and autonomy due to financial independence. Furthermore, it also affects one's social bounds within and outside of the workplace (15). Volunteering is even recommended to elderly people after retirement to retain relationships (16). Since people with disabilities are also underrepresented in professional life, volunteering is an alternative to a regular job or working at a social service provider. Volunteers engage with the community and pursue meaningful activities. Kruithof et al. (17) confirm this after interviewing people with disabilities who volunteered as hostess, barista, football equipment manager, gardener or craft worker in the Netherlands. According to the participants, their occupation brought structure to their daily lives. They enjoyed their work and thus, accepted not receiving any money for their work. The volunteers with disabilities also noticed a change in interactions with people in their community who recognized them. It made them feel like being part of this group since presuppositions surrounding disabilities were reduced (18). Thus, they bonded with strangers and even formed relationships with them. Those positive experiences contribute to increasing the chances of employment (14). The volunteers develop new social and mechanical skills and as a result, their self-confidence increases. A person without work experience as a volunteer takes significantly longer to find a job (19). Thus, people with disabilities benefit from this arrangement, especially since only about half of the people with disabilities are included in the job market in the EU whereas approximately 75% of people without disabilities in the EU pursue a job (20). In the U.S., for instance, this gap is even more significant as there is a difference of 34.4% between the employment of people with and without disabilities (21).

Some volunteers seem to be aware of those benefits which could be a reason why they engage in this field. Yanay-Ventura (22) investigated what motivates people with disabilities to volunteer for different causes. While the motivation for some is to be active and help people, others want the work experience as well as to meet people and pay their community back for the support they received. However, the participants notice that their abilities are regularly underestimated and their roles do not fit them well. In order to find suitable jobs for the individuals, Yanay-Ventura (22) demand that volunteer organizations provide a coordinator to support people with disabilities and ensure accessibility at the respective locations. Those additional expenses as well as beliefs about challenging behavior, difficulties complying with unwritten rules at work, lack of work experience and low levels of literacy that concern people with disabilities (14) might be reasons for volunteer organizations to refrain from pursuing an inclusive approach.

Nevertheless, several volunteer organizations are willing to address those challenges and find solutions to work with people with disabilities. Wicki and Meier (9) describe the situation in Switzerland where volunteering companies develop communication strategies for volunteers with disabilities to overcome any potential difficulties at their workplace. According to Cheah et al. (23), this support system assists people with disabilities in finding a job and getting to keep it. Thus, meeting the volunteers' needs should be facilitated by assessing their skills correctly and understanding their reliability when working. For instance, they get more time to complete their tasks which reduces their workload. Yanay-Ventura (22) interviewed volunteers with disabilities who took on occupations as seminar presenters, management staff or teachers within an Israeli organization. They attended internal training with colleagues and participated in courses to prepare for their tasks. As an effect, hardly any negative volunteering experiences were reported.

Generally, it is important for the organization to adjust their proceedings when cooperating with people with disabilities during an event according to their individual needs. Kappelides and Spoor (24) stress that flexibility and empathy portray a key strategy to create an acceptable work environment. Undlien (5) investigated the collaboration between a Norwegian school and the organizers of the 2016 Youth Olympic Winter Games. Interviews about the collaboration were conducted with students with disabilities who worked as volunteers, their teachers who supported them in terms of the organization of their job as well as the event's head of volunteers. The assigned task for the students with disabilities was picking up litter at the event. Some of the teenagers deemed this activity as helpful for saving the planet. All volunteers enjoyed their task and being part of the sport event. Despite some challenges which included the electronic registration that required assistance and some distraction from their work such as talking to others, the teachers, the head of volunteers and students with disabilities were satisfied with the overall process. The head of volunteers as part of the event organization team especially enjoyed the cooperation for the students' enthusiasm and reported that the challenges were similar compared to other groups from schools. Solely, finding the appropriate task required more effort in order to promote its meaningfulness. At the same time, the students also had to be able to perform the task without facing major difficulties. This cooperation highlights that including people with disabilities on the team of a sport event is even feasible and meaningful, if a supporting party for the volunteers with disabilities such as the teachers in this study (5) is established. In addition, other studies highlight the importance of a support system as well (25).

Another advantage for the organizations with people with disabilities is that social acceptance takes place as the volunteers with disabilities establish relationships with other volunteers (24). Working alongside in a similar uniform creates a feeling of belonging and equality among all volunteers (5). Thus, prejudice against people with disabilities can be dispelled which can be explained with the contact hypothesis (18, 26). It illustrates how “bias or negative attitudes toward a group can be reduced through purposeful contact that includes cooperation and equal status between groups within a supportive environment” [(27), p. 88]. This can elicit a more positive attitude towards people with disabilities (28). The volunteers with disabilities also serve as role models for other individuals with disabilities (24). Miller et al. (28) even noted higher levels of productivity and efficiency in volunteers with and without disabilities in mixed-abled teams.

### 1.2 Educating volunteers as sport management assistants with disabilities

Training courses are an option to prepare people with disabilities for their tasks as volunteers (22). In the sport event sector, training courses for becoming a volunteer are also a viable method for people with disabilities. Evidently, designing those courses is difficult as there persists a lack of addressing the diversity of society in sport. McDowell, Pickett, and Pitts (29) showcase this absence of diverse topics in the U.S. sport management sector and demand more representation in sport management education. Only a minimal amount of less than one percent of literature is related to disability sport, leisure, recreation, or physical activity (30, 31). Among other reasons, this might explain why the topic of disability is hardly discussed during introductory sport management courses at universities (32).

Sport management courses designed especially for people with disabilities are rare. To redress this problem, a sport management course for instructing volunteers with disabilities has been initiated in Graz, Austria by a social service provider in 2021. The non-university training seminar is based on frameworks concerning inclusive community education, enabling social inclusion in organizations and sport (4). It entails a theoretical (11 sessions) and a practical aspect (8 sessions) which amounted to 184 units in total (one unit equals 45 minutes).

In order to enable their employment at regular sport events, the sport management assistants discuss topics such as organized sport in Austria, inclusion through sport, communication as well as sport event management which surmounts to one third of the course content. Their timetables include teambuilding, establishing self-confidence, stages of project planning of an inclusive sport event or the UN Convention on the Rights of Persons with Disabilities (1). In cooperation with Special Olympics Austria and the Austrian sport governing bodies, the participants also master practical training such as observations, sitting in on various sport events, assisting during those events and reflecting on one's experiences which constitute half of the sessions in the course curriculum (see Table 1). According to Shapiro and Pitts (30), the design of this course can be defined as an infusion model since it requires the attendees to ponder theoretical input and establish a connection to the practical aspect of sport event management. It can be compared to other sport management courses, for instance, at universities which also rely on the infusion model [cf. (33)]. The remaining 20% of the training course are spent working on a final project in order to graduate from the program. Ideally, the participants realize their own grassroots sport event and document the concept as well as the execution of their idea (34).

**Table 1:** Content of the training course for people with disabilities.

Kind of input	Topic	Units
Theory (58 units)	Teambuilding	8
	Inclusion	5
	Sport in society	5
	UN Convention on the Rights of Persons with Disabilities	5
	Inclusion in sport I & II	10
	Project management	5
	(Sport) Event management I & II	10
	Communication	5
	Conception of the final project	5
Practical training (126 units)	Observation	12
	Shadowing during event	12
	Assisting during event	36
	Reflection	24
	Final project	6
	Realization of the final project	24
	Documentation of the final project	12

### 1.3 Research Questions

Studies have acknowledged that people with disabilities are hardly included in sport event organization and highlight a lack of training opportunities to work as volunteers or sport management assistant (2, 24). Based on the findings of Kappelides and Spoor (24), the inclusion of people with disabilities at sport events is an advantage for all employees at the event as well as the sport event organizers. However, there persists a lack of people with disabilities on the management teams of sport events. Therefore, the aim of this study is to investigate the development and implementation of the cooperation between sport management assistants with disabilities and sport event organizers in Austria prompted by a training course. Four research questions serve as the foundation of the study:

1. How do the sport management assistants with disabilities benefit from their training course to prepare them for working at sport events?
2. Under what circumstances do sport management assistants with disabilities benefit from working at a sport event?
3. How can the cooperation of sport event organizers and sport management assistants with disabilities be facilitated?
4. What encourages sport event organizers to include sport management assistants with disabilities in their events?

## 2 Materials and method

To answer the research questions, the sport management assistants with disabilities participated in a qualitative interview to evaluate the theoretical contents of their course as well as their participation as volunteers in sport events. Next to the sport management assistants, the organizers were also interviewed based on their experience in the collaboration with the sport management assistants. This allows a thorough understanding of the structure behind the training course as well as the sport events that the sport management assistants worked at.

### 2.1 Participants and procedure

The research project entailed interviewing a total of eleven people (7 female and 4 male) over a period of three months from December 2022 to March 2023. Out of a total of ten course participants, six sport management assistants with disabilities (4 female and 2 male; age *M* = 31.67; *SD* = 6.07) as well as five sport event organizers (3 female; age *M* = 41.2; *SD* = 9.04) were selected for the interview process. The former group has been involved with sport before starting their training. The latter group was contacted through email and qualified for the interviews because of their involvement in the sport events that the assistants with disabilities were employed for and their experience based on the events. They are experienced at organizing sport events as their work experience ranges from three to 26 years (*M* = 10.4; *SD* = 8.52) and two out of the five event organizer interviewees have been engaging with people with disabilities for numerous years. The disciplines at the events comprised track and field, running, climbing and ice hockey. The length of the interviews varied from nine to 26 minutes (*M* = 16.82; *SD* = 4.97). The majority of the interviews was conducted in person (*n* = 10) while one of the event organizers was interviewed by phone for reasons of practicability.

### 2.2 Interview guide

Since two different groups of participants were included in this study, two interview guidelines with similar content aspects were created. They are mostly based on a study by Kappelides and Spoor (24) who consulted volunteers with disabilities as well as organizing staff and participants in the event in order to determine manners of supporting volunteers with disabilities. They highlighted advantages and barriers of including volunteers with disabilities from three different perspectives. Those aspects were particularly helpful for establishing categories for the analysis of the interview data such as the motives of participating in or creating an event which, according to Kappelides and Spoor (24), range from social acceptance to social inclusion and personal development. Overall, 11 different categories were established, namely, training, motives, task allocation, payment, desires and prospects, collaboration, event organization, payment, benefits and barriers. In the interview, questions such as “What is the event that you organized all about?” and “What was your personal experience that you gained from participating in the sport event?” were asked. This feedback becomes more detailed by including the research of Yanay-Ventura (22) who interviewed volunteers with disabilities working in different fields and asked for their roles in the sport event. Concerning this category, the sport management assistants with disabilities and sport event organizers responded to questions such as “What were your responsibilities at the events?” and “How was the contact with the sport management assistants during the event?”. In the aforementioned study, the interviewees were also inquired about their positive and negative experiences. The interview guide introduced this issue by asking “What is your impression of the event management team?” and “Is there something about the event that you did not enjoy?”

The category desires and prospects is also based on the study by Kappelides and Spoor (24) as all parties surveyed by the researchers identified barriers concerning negative attitudes, organization and lack of social inclusion that are detrimental to inclusion of volunteers with disabilities in sport events. Moreover, Yanay-Ventura (22) asked for future personal goals of the participants. During our interviews, the event organizers and sport management assistants had to answer questions such as “Do you have any ideas how to improve the event on future occasions?” and “What do you plan on doing with your training as sport management assistant?” The final category of payment is discussed by Kruithof et al. (17) whose research showed that volunteers were content with their job and did not demand any financial compensation for their efforts. The interviewees in this study were asked “How much money did the sport management assistants receive for working at your event?” and “Do you think the amount of money you received for working at the event was sufficient?”.

The questions of both groups were divided into sections discussing those varying topics. Some differences in regard to the content of the categories existed between the interview guidelines. One of them concerned the training course of the sport management assistants since they also had to comment on their experiences prior to the sport events. The interview guide for the organizers also differed from the other one as they had to touch upon the topic of inclusion of people with disabilities in society through their engagement in sport events and advantages and disadvantages of working with people with disabilities at their event.

### 2.3 Data analysis

After recording and transcribing the interviews, the transcription was proofread in person for errors. The qualitative content analysis (35) provided the foundation for the data analysis. First of all, the coding procedure was established deductively based on the theoretical groundwork (17, 22, 24). For statements that were impossible to sort into any of the pre-existing categories, inductive categories were created. Afterwards, the distribution of the interview sections to their according categories was discussed in the research team as part of a consensual process. Thus, the analysis follows the categories hereinafter. For the sport management assistants with disabilities the five aspects training, motives, task allocation, payment as well as desires and prospects are relevant for the analysis. In terms of the event organizers, the five factors collaboration, event organization, payment as well as benefits and barriers were regarded. Since the two groups of interviewees had different experiences leading up to the event, this differentiation was deemed necessary. The category payment is approached from both points of views.

## 3 Results

### 3.1 Sport management assistants with disabilities

#### 3.1.1 Training

In general, the sport management assistants commented positively about their training course after completing it. In the interviews, the participants shared many positive impressions from the training course. All interviewees would recommend it to people who enjoy doing sport or like working in a team. Throughout the interviews, three aspects in particular were apparent that impacted the group on a deeper level. First of all, the team spirit among the sport management assistants was profound. Over the course of their training, the assistants became a cooperating group. They were on good terms with each other. The participants also stuck together and worked well in their team, even when they were stressed. If there was a problem, they were able to speak openly to one another:

I really enjoyed collaborating with the other members of the training course. We worked really well as a team. Sometimes, we joked with each other but all things considered, we grew into a really good team. (B1, 00:08:04)

Next, the assistants also gained knowledge related to sport event management that they were not familiar with at first. They realized what considerations are necessary in event planning:

What I really liked about the training was that you got to know in detail what is actually needed to execute a sport event. I did not know that beforehand even though I am an avid fan of different kinds of sport and attended sport events in the past. I just never realized all the things you have to consider. (B3, 00:00:34)

One of the sport management assistants with disabilities is now part of an inclusive sport club where she helps with event organization:

I help with different sport events now. Well, yes, I help with an inclusive sport club already. Whenever there is an event, I help out. For instance, with the swim competitions, I am part of the organizing committee. (B2, 00:06:26)

However, she stresses that she would not organize an event by herself but that she requires support from others. Similarly, another interviewee recognises the advantage of working together in a team when organizing an event at a bigger scale since the team partners help with remembering tasks. Another participant is able to perceive bad event organization observed in their free time. Apart from the theoretical background surrounding sport event organization and the reflection of their experiences at several sport events, the group eventually created their own sport event as part of their final exam. This last task of the course was a group effort and the assistants hosted a bowling competition in October 2022. Individuals in their team held different roles and one of them said that the job as tournament director was not exhausting at all but important.

Thirdly, the sport management assistants with disabilities also perceived a change in their everyday lives because of the training course as one of them depicts:

Yes, now I am considerably more in touch with other people, my free time is filled with different activities now, in the afternoons I have got something to do and I have more of this free time and that is just fantastic. (B1, 00:04:20)

Another participant noticed a change in her personality as she claims that she is now able to cope with stressful situations.

I learnt to deal with stressful situations in a considerate manner. Of course, I was able to manage those situations beforehand, as well, but during the course I somehow learnt to get rid of the pressure in certain circumstances. (B4, 00:01:27)

Those positive impacts on their lives might account for the assistants' interest in taking part in more events. One person even wants to continue working without taking a break.

#### 3.1.2 Motives

The sport management assistants with disabilities have different reasons for engaging with the respective sport events. Their main motivation for attending the course was their passion for sport as they can be active and part of a group. In addition, physical activity, improving in their sport and getting to know new skills were other motivational factors to enrol in the training course. Their answers changed slightly throughout their training as sport management assistants. When asked for their motivation during the interviews, working alongside other people and getting to know them once again made the list of motivating aspects at sport events. One sport assistant loves working with people with and without disability:

I really enjoy the collaboration with other people. I love getting to know new people. Well, yes, I just like to collaborate with the people, but not only with the ones with a disability but also the ones without one. (B5, 00:06:09)

But the interviewees also referred to motivating experiences that had not been addressed before. One person appreciates the food and drinks they receive at the events, while another one was delighted to ask mascots at an event to take a picture together. However, their personal gain is not the only motivation for the assistants. They also seem to recognise the significance of their presence at sport events for society as the following quote highlights:

Just the inclusion at the events we were part of. This is not just about one event for me. For me it is more important to show people “hey, a person with disability may sit anywhere they want to” (B3, 00:09:27)

In addition, the majority of sport management assistants with disabilities recall that the event organizers praised them for their work at the various sport events. This seems to be another motivating aspect.

#### 3.1.3 Task allocation

Finding the right tasks for sport management assistants with disabilities is a crucial part of a successful collaboration. One participant highlights the importance of the group leader who stayed in touch with the event organizers and chose the right tasks for everyone in the group. The assistants had a wide range of tasks to cover during the different events:

We helped with admittance and the registration of people. […] We worked all day long from 11 am until nearly 8 pm. […] This time, we also helped with admittance. We distributed drinks as well as medals. […] For most of the events, we helped with the setup. (B1, 00:00:18)

In addition to admitting and recording athletes, the assistants also had to register journalists. On several occasions, they handed out medals and refreshment to the athletes. One time, more than 7000 medals had to be given to the athletes. Another participant also recalled this situation and was excited to see how the exhausted athletes crossed the finish line. One participant remembers having to rip packaging in order to hand two water bottles to the runners. While this experience is rather unnerving for some assistants, others enjoy the busy atmosphere at the events and keep track of the situation.

#### 3.1.4 Desires and prospects for their work

In the interviews, the participants answered questions about their ideas for future events and the issue of accessibility was touched upon. Long periods of having to stand in one place was affecting one interviewee. Especially standing on asphalt for several hours contributed to back pain. But one of the sport management assistants had the following solution:

[…] We need a bigger team. Then we can say “let’s send five or ten people”. So, an operating cycle is set up. One person works for two hours, then there is a 15-min break and someone else takes over. This goes on and on. I would enjoy that a lot. (B5, 00:07:43)

For this idea to work, the organizers would have to employ more people or redistribute people engaged with other tasks to fulfil this need of the sport management assistants. Another sport assistant with disability complained about a children's only contest at the sport event. The children could visit those stations and complete different tasks but it was not well-frequented and the participant was bored.

The interviewees also talked about their personal future endeavours because of the course. A participant says that it could indeed be her dream job to contribute to sport events.

I want to attend a lot more sport events. Maybe it could even be my dream profession later in life. Because at the moment I do not know what my dream job could be, but maybe it could be that I am tied in a related line of work and then I can contribute to the events. (B1, 00:09:11)

### 3.2 Sport event organizers

#### 3.2.1 Collaboration with sport management assistants with disabilities

The reports of the sport event organizers about the collaboration with the event team were quite positive. In three out of five cases, the group instructor approached the organizers. For her track and field event, one organizer wanted to engage people with disabilities and received contact information of the group leader. Similarly, another organizer was adamant about including people with disabilities in the event staff:

This is an important topic for me personally. I think you need the right person in your company that sees the importance [of inclusion]. While it is important to me, it is also part of our business culture since we like these [inclusive] aspects. Also, the event can only take place if there are enough employees. […]. If there are people willing to work and want to be part of it all, we are certainly not refusing them. (B8, 00:03:16)

The organizers were satisfied with the sport management assistants and gained a positive impression of the team. Their motivation during the setup of the event was highlighted as well as their ability to learn. The assistants were already familiar with their tasks and needed fewer explanations than in the beginning. They were in high spirits and displayed enthusiasm for the athletes. Despite some organizers not directly being in touch with the assistants during the event but their coordinators, the feedback for the group was positive. As a consequence, they already worked at one event several times. The role of the group leader who supported the team if they needed him was also pointed out. The sport management assistants also proved their worth to the coordinator at an ice hockey event and an organizer admits that his event organizing team underestimated the group's abilities:

[The coordinator] said that the process at the event was very smooth. I think that we can include them more in an upcoming event. We probably underestimated them. We saw that they could cope with more responsibility and a greater workload. (B10, 00:02:58)

#### 3.2.2 Event organization with sport management assistants with disabilities

The event organizers were aware that the assistants needed detailed explanations of their tasks. Thus, it was necessary to consider carefully which tasks to assign to the group. One event venue lacked accessibility due to several staircases. The only suitable job entailed the preparation of snacks for the crew at the event. The remaining organizers did not have to comprise due to accessibility issues.

The tasks depended on the nature of the event. At the indoor climbing event, the event group covered organizational tasks such as admittance, checking the attendance list and distributing name tags. They also set up the climbing gym by arranging tables and flyers, banners or bow flags. The buffet was also part of their responsibility. The jobs at an ice hockey rink were fewer since the assistants had to place towels, hand out pucks to athletes and press passes to journalists or greet VIP guests. The tasks during an outdoor running events did not differ from each other and involved preparing refreshment for the athletes as well as putting finisher medals on them. Concerning the task allocation, the insecurities of organizers who collaborated with people with disabilities for the first time were prevalent. The lack of experience regarding the job assignment also concerned the company of one interviewee. Those worries turned out to be unfounded and, hence, the company continues to hire sport management assistants with disabilities. One person provided a solution for the issue with missing knowledge about the collaboration:

I think that one needs more information in advance about what it means to promote inclusion at your event. Naturally, guidelines and to-do lists exist but that is not always applicable to our events. […]. There is a lack of knowledge and time to engage thoroughly with the topic. You need someone to contact who visits you for an hour and tells you five things that are easy to implement and do not multiply the expenses. (B8, 00:16:45)

Another interviewee, who confirmed that the inclusive tendencies were enhanced by the cooperation with the event group, suggested a similar concept with a contact person as well as someone on their own team to engage with sport clubs and individuals interested in competing at the sport event. Another organizer considered extending their initiative to schools and including a wheelchair race at their event in order to reach a wider audience. Consequently, inclusion for the sport management assistants and for athletes at the event could be accentuated.

#### 3.2.3 Advantages of the collaboration

All event organizers stated positive impacts of including sport management assistants with disabilities. They witnessed favorable developments for their own event such as better vibes among the attendees at the event. The enhanced image of the event due to the cooperation with the event team was also mentioned but it was stressed that this was just a secondary effect. The presence of the sport management assistants with disabilities enriched the events and one of the event organizers even initiated a report on the local TV station to spread the news of the successful cooperation. Similarly, another interviewee wanted to prove to attendees of the event and other event organizers that it was possible to work together with people with disabilities. Consequently, people who were less prone to experience inclusion in their everyday lives came into contact with people with disabilities at the climbing event.

The impact of the event on the sport management assistants with disabilities was also discussed. What proved to one event organizer that his event meant a lot to the sport management assistants was the shirts they received at the event as they continued wearing them several weeks afterwards. Another interviewee reckoned that any assistant regardless of their role at the event benefited from the experience in sport event management. Her company attempts to let their staff have some sense of the responsibility that accompanies sport event organization. Consequently, the people involved in those events benefit from their advanced work experience. While it was recognized that some assistants refined their work routines at the recurrent climbing event over the course of several sessions, a person referred to the significant contribution of the assistants to the event:

It is important to us that everyone is wanted. It does not matter which qualifications we have to work with. The staff has to be appointed to a job that is the right fit for them. Everyone brings along attributes we need and I very much hope that they [the sport management assistants] saw their great importance for the event and that they benefited from that. (B7, 00:07:32)

One of the most important aspects about the collaboration with the event team was the lasting societal impact. The event organizers were aware that people with disabilities are hardly in the public eye. Especially when it comes to sport, they are underrepresented. One sport event focused on conveying the importance of health promotion among people with disabilities and to present climbing as an activity for staying active. It also raised the public's awareness about disability which is noticeable in climbing where a connection between the climbers is established due to the social nature of the sport:

If people who have never heard of the event are in the climbing gym before or during our event takes place, they usually will join us, ask questions about our business and they are curious […] and they are open and might say, “Sure, I will belay and show something to someone.” (B9, 00:16:45)

Similar tendencies were identified by another organizer who named two major influences of his event on the public. One the one hand, people with disabilities had a way of identifying themselves as passionate about sport and actively engage with it as assistants. On the other hand, people without disabilities learnt from them:

I think that people with disabilities are more appreciative of everything compared to other people. Because of this, we can learn so much from each other since one person might be concerned with a totally insignificant problem. You have to make a lot of people aware of the implications of disabilities […]. This is a learning curve for society. (B11, 00:09:07)

In this regard, he also emphasized the key role of the media in the domain of sport as people who did not attend sport events can still grasp a sense of the inclusion in society. Another event organizer aimed at making coexistence in sport possible regardless of people's abilities or performance. She deemed separation of otherness detrimental to inclusion and wanted to achieve solidarity in society. In the long run, inclusion in sport benefits everyone in society:

A lot of things do not fit together here. It is sad that it is dependent on a politician's mindset. This [inclusion] should not be politically determined. Because if you plan ahead, all of this is prevention of one's health and well-being. If you do sports, you are different. You are more motivated and focused. […] An athlete's goal is to be fit enough for their competition on the weekend or their training during the week. (B7, 00:19:51)

#### 3.2.4 Barriers of the collaboration

It was impossible for the event organizers to imagine their events without the sport management assistants with disabilities being part of it. However, the organizers confessed to some barriers during their cooperation with the event group. The accessibility of the venue was problematic. One organizer admitted that someone using a wheelchair would have struggled during their outdoor event if it had rained since the softened ground would have been difficult to handle. Kerbsides, cable bridges and various forms of ground such as grass and concrete posed another problem with accessibility which also affects indoor events. The climbing gyms had to be inspected to suit the needs of people with disabilities. Narrow hallways and too many staircases were exclusion criteria for potential venues.

Nevertheless, the inclusion of sport management assistants with disabilities demands the adaptation of usual processes. One interviewee explained that they received more support of a coordinator than assistants without disabilities to ensure the success of the running event, especially since the participants of the event had to pay a fee to attend the event. One organizer also invested more time when working with sport management assistants with disabilities and adjusted her working schedule to meet their needs:

What one has to consider right from the start is easy and distinct explanations and maybe they need to hear them twice or three times. But you simply take more time in the beginning. I always meet up with the team before the event and discuss their jobs before people arrive […] you simply take a bit more time […] and take 15 min for a different explanation in an easier language. (B9, 00:08:30)

Despite the extra effort in terms of accessibility, personnel or time resources, the event organizers were aware of their responsibility concerning inclusion. One participant stressed that being different does not define a person but that one's positive attitude is the crucial factor for the success of a sport event. Another interviewee wanted his crew at the event to take their job seriously and to communicate with the athletes. In his opinion, the sport management assistants did just that. They were enthusiastic about the athlethes' performance and grew with the challenge. Moreover, they encouraged fellow event organizers to realize the cooperation:

You learn things and it is no use to be afraid of mistakes. […] Apparently, some event organizers believe that by refraining from this cooperation they avoid doing something wrong. That is not our mindset. (B9, 00:18:22)

### 3.3 Sport management assistants and sport event organizers about payment

According to the assistants, the event organizers handled the financial matter differently. At one event, all supporters received a 20-euro-voucher. On the contrary, one event organizer did not pay them any money in the first year of their collaboration. After reflecting on this with the course instructor, they realized that the assistants work “as all the others do” (B8). In another instance, the group leader sent an invoice to the event organizer. The payment is transferred to a bank account where a month's earnings from various events is collected before paying the individual members. One event organizer wanted to establish equality within the staff as the sport management assistants received the same amount of money as other staff members with similar tasks. In one instance, the interviewee did not know whether the event management team paid the event group at all but agreed that the assistants deserve to get paid for their work.

The assistants seemed to be satisfied with the current arrangements. One participant was happy about the experiences that she gained from working at the event and did not wish for a higher salary. Another event group member had a different outlook on this matter:

I am such a big fan of sport events that I would actually say that I could work at the events for free and not receive any money but generally, I am happy with the arrangements. (B3, 00:08:56)

## 4 Discussion

The present study surrounding a training course for sport management assistants with disabilities and their subsequent employment at sport events covers the viewpoints of those assistants as well as the event organizers they worked with. The topics training course, motives, task allocation, payment, desires and prospects as well as collaboration with sport management assistants with disabilities, event organisation, barriers and advantages are amplified by the interviewees. One of the main pillars for incorporating the assistants of this study in the organization of sport events was their specialized training that spanned over several months and prepared them for their occupation at the various sport events in different manners. Generally, the training course for sport management assistants with disabilities is another valuable opportunity for inclusion in sport (36). It provided the participants with sufficient theoretical and practical input surrounding sport event organization. The graduates from the course were satisfied with it and praised the team spirit which was promoted by teambuilding activities. Establishing those interpersonal connections serves as a social resource which is indicated by some of the assistants withstanding stressful situations as they relied on their group's support and thus, buffered stressors (37). Other instances of encouragement of peers can also be traced in the assistance of their mentor leading their training course or the superiors at the sport event who cared for their needs during the occupation at the sport event (25). This social connection underlines one of the main motives for participants of the event group to complete the course (22).

The practical aspect of their training was especially exciting for the sport management assistants with disabilities which became apparent during the reflections of their various roles at the sport events. Those experiences left a perceptible impression with them as they vividly recalled memories from the events that appeared to have a positive effect on their self-esteem and self-efficacy (24). This development could be due to their ability to physically and mentally cope with the challenges at the events which positively impacted their mental health (38). Moreover, the assistants could show other people what they are able to achieve and thus, refuted ableist societal norms (39). Concerning the financial aspect, the participants were satisfied with the arrangements, albeit not receiving money at one event. In fact, one participant would even work at the events without receiving any money since he enjoys sport. Notwithstanding, one organizer wanted to create equality within his team working at the sport event and equal pay is a viable option for this undertaking (40). One of the course participants was quite enthusiastic about working at the event and she even considered sport event management as a career option.

It is vital to make the events accessible for people with disabilities as physical and organizational barriers prevent their inclusion. Issues with accessibility are even conspicuous at major sport events, which do not cater the needs of people with disabilities especially regarding service delivery (41). Collaborating with people with disabilities could draw the attention of event management teams to accessibility. As one of the sport management assistants suggested the employment of a greater amount of people in future events is helpful for establishing an operating cycle which allows regular break intervals and prevents him from meeting his body's limits. Despite some organizers remarking on the lack of sufficient financial resources, they also agree with the notion of adequate working conditions for their employees to keep up their morale and conduct a successful event. Furthermore, the organizers have to be considerate of sport management assistants with disabilities when communicating with them. One event organizer emphasised the need for intelligible explanations which should be repeated several times. As mentioned by one of the interviewees, the extra expense on personnel and accessibility are discouraging for many event organizers who, hence, refrain from including people with disability on their staff and “entrench nondisabled cultural norms” [(25), p. 2]. A mediator such as the group leader of the study's training course who facilitates the communication between the event organizers and the sport management assistants with disabilities by, for instance, finding the appropriate task at the event, can bring about a change in already established settings and elevate inclusion in the domain of sport (42).

Pursuing the collaboration with sport management assistants with disabilities was worth the effort to the sport event organizers because of the event group's enthusiasm which also positively influenced visitors and staff. Paradis et al. (43) show that inclusive and disability-specific sport events such as parasports increase the number of people with disabilities doing sport. They also contribute to enhancing the public's awareness about disability and a positive change in their attitudes. The mindset of staff who work together with people with disabilities is more favourable towards their colleagues as they gain an understanding for them (44). Moreover, those positive outcomes reduce the number of hesitant event organizers to collaborate with people with disabilities (28). The sport event organizers interviewed highlighted that there are barriers such as lack of support leading up to the event for their colleagues in event organization which prevent the collaboration. Consequently, an external consultant could be helpful to provide event organizers with advice and guidelines to alleviate their concerns about the sport management assistants.

Overcoming those hurdles is of utmost importance since once the collaboration was established the organizers of this study spoke highly about their experiences. Some of them even want to allow for more inclusion at their events after their cooperation with the event team. One event organizer plans to incorporate a wheelchair competition at the event, while another one will attempt to approach schools to reach a wider audience. Thus, the inclusion of sport management assistants with disabilities at sport events encourages the sport event organizers to make sport events more inclusive in general, which is a positive consequence of including people with disabilities in the organization of sport events and should be addressed in terms of the diversity in volunteers and volunteer management (45).

### 4.1 Limitations

Naturally, this preliminary study only represents a small population in a specific region. The qualitative research method only superficially covered aspects of the collaboration. For instance, the sport management assistants with disabilities were not asked about their opinion regarding the cooperation with individual event organizers, while the latter commented on the performance of the event group. However, this study gives a first insight into the education of sport management assistants with disabilities and their collaboration with sport event organizers.

### 4.2 Implications for further research

The sport management training course allows people with disabilities to engage with sport event organization. It is beneficial to their personal development as they receive approval for their work, establish social connections as well as overcome difficult situations which nurtures resilience. The successful integration of sport management assistants with disabilities at the events depends on meaningful tasks and fair working conditions such as an accessible workplace and appropriate language. In this regard, a mediator who represents their interests to the event organizers is significant. However, the event organizers also depend on a person knowledgeable about the topics of creating a good work environment for people with disabilities or regulations regarding accessibility to receive information for approaching the collaboration with the sport management assistants. The inclusion at the event also impacts the attitude of other people involved with the event such as staff and spectators towards people with disabilities positively.

In order to gain a deeper understanding of the arrangements, focus group interviews featuring both parties involved could provide a bigger picture and fill the gaps in knowledge. It would also be beneficial to interview athletes competing at sport events and inquire about their impression of the sport management at the event. That could be especially insightful since a majority of the athletes could compare their experience with events that did not employ volunteers with disabilities. Furthermore, there persists the need for more research with participants in diverse sport event settings in different countries and group constellations to promote inclusion in sport. Intervention studies could be implemented to display the firsthand effects of including volunteers with disabilities on all parties concerned with the sport event.

## Data Availability

The raw data supporting the conclusions of this article will be made available by the authors, without undue reservation.
